# 175 FunFit: A Collaborative Community-based Physical Activity Programme for Children aged 6-12 with Diagnosed Delayed Gross Motor Skill Development

**DOI:** 10.1093/eurpub/ckae114.014

**Published:** 2024-09-26

**Authors:** Clare Deasy, Eoin Kaar

**Affiliations:** Cork Kerry Community Healthcare, Health Service Executive, Cork, Ireland; Cork Sports Partnership, Cork, Ireland; Munster Technological University, Cork, Ireland

## Abstract

**Main Purpose:**

FunFit is a FREE community based physical activity programme for children aged 6-12 with diagnosed delayed gross motor skill development. The programme was developed in response to a gap identified by paediatric therapists around a lack of appropriate community programmes to signpost children attending their services to. FunFit aims to provide a physical activity opportunity in a fun, inclusive, non-competitive environment to build not only movement skills but also confidence & self-efficacy around being active.

**Project Description:**

Development: FunFit was developed by the Cork Kerry Community Healthcare Health Promotion & Improvement Physical Activity team in partnership with HSE primary care Paediatric Physiotherapists & Cork Sports Partnership.

**Implementation:**

FunFit is a group activity programme offered in blocks of 8 x 1hr weekly sessions, held in community-based facilities in Cork. Sessions are delivered by appropriately qualified and vetted exercise professionals who have attended specifically developed FunFit Tutor Training. Access to the programme is by paediatric healthcare professional referral only. Referrals are co-ordinated by the Health Promotion & Improvement Department whilst Cork Sports Partnership co-ordinates tutor and venue requirements.

**Evaluation:**

A subset of children formed part of a pre & post intervention evaluation.

PA levels remained low post intervention, yet an upward trend in mean days of PA per week was observed (4.31±1.55 versus 3.69±1.6).

In intervention group 2 (N = 9), statistically significant improvements were found post-intervention in total locomotor (P = 0.012, effect size (ES) = 0.589), total object control (P = 0.034, ES = 0.499) and total FMS (P = 0.008, ES = 0.629). A statistically significant increase in mean days of PA per week was observed (4.33±1.55 versus 3.25±1.76). A positive improvement in enjoyment of PA (P = 0.014, ES = 0.502), and self-efficacy (P = 0.035, ES = 0.430), were also reported post-intervention.

**Dissemination:**

A key goal for 2024 is to expand FunFit to Kerry as well as prioritising areas of deprivation in Cork, where need is greatest.

**Conclusions:**

FunFit has demonstrated the importance of interagency collaboration & referral only programmes when trying to engage children who are at risk of low lifetime physical activity participation.

